# Worldview Under Stress: Preliminary Findings on Cardiovascular and Cortisol Stress Responses Predicted by Secularity, Religiosity, Spirituality, and Existential Search

**DOI:** 10.1007/s10943-020-01008-5

**Published:** 2020-03-27

**Authors:** Tatjana Schnell, Dietmar Fuchs, René Hefti

**Affiliations:** 1 Institute of Psychology, University of Innsbruck, Innrain 52, 6020 Innsbruck, Austria; 2 MF Norwegian School of Theology, Religion and Society, Oslo, Norway; 3 Division of Biological Chemistry, Biocenter, Innsbruck Medical University, Innsbruck, Austria; 4 Faculty of Medicine, University of Bern, Bern, Switzerland; 5 Research Institute for Spirituality and Health, Langenthal, Switzerland

**Keywords:** Trier Social Stress Test, Religiosity, Spirituality, Atheism, Existential search

## Abstract

This study reports preliminary findings on the hypothesis that worldview can predict cardiovascular and cortisol responses to social stress. Based on theory and previous findings, we assumed that worldview security would provide a basis for stress resilience. Accordingly, religious and atheist individuals were expected to show higher stress resilience than spiritual and agnostic participants. Likewise, dimensional measures of religiosity and atheism were hypothesized to predict decreased, and existential search—indicating worldview insecurity—was hypothesized to predict increased physiological stress responses. Subjects included 50 university students who completed online questionnaires and took part in a standardized social stress test (Trier Social Stress Test). Systolic and diastolic blood pressure (SBP/DBP), heart rate (HR), and salivary cortisol (SC) were assessed at baseline, immediately after stress testing, and during a forty-minute recovery period. Worldview comparisons revealed lower cardiovascular stress responses among religious than among atheist and spiritual participants and particularly high baseline SC among spiritual participants. Across the entire sample, existential search showed substantial positive correlations with SBP, HR, and SC stress parameters. The findings suggest that worldview security might partly explain the health benefits often associated with religion.

**Electronic supplementary material:**

The online version of this article (10.1007/s10943-020-01008-5) contains supplementary material, which is available to authorized users.

## Introduction

Studies have repeatedly established positive links between religion, spirituality (R/S), and health. This pertains to self-rated health (e.g., Green and Elliott 2010; Headey et al. 2014) as well as to objective measures of health and illness, such as cardiovascular functioning/coronary heart disease, Alzheimer’s disease, dementia, immune function, onset, outcome, and recovery from cancer, recovery from stroke, spinal cord injury, and HIV infection (Hemmati et al. 2019; Hill and Pargament 2008; Ironson et al. 2006; Johnstone et al. 2008; Koenig 2012; Koenig et al. 2012; Matheis et al. 2006; Powell et al. 2003; Saad 2019; Vance et al. 2008). Lastly, and probably most impressively, several studies have confirmed that higher R/S is related to greater longevity (Chida et al. 2009; Idler et al. 2017; McCullough et al. 2009).

The terms “spirituality” and “religiosity” are often used interchangeably. Nevertheless, most of the above studies operationalized “R/S” by use of elements of traditional religion, such as belief in god, prayer, and religious service attendance. But spirituality has recently turned into a worldview that can be distinguished from traditional religion. While some people call themselves spiritual as well as religious, others use the term spirituality to express a distance from religion. It thus becomes an alternative worldview—or rather an umbrella term for many different worldviews (Schnell 2011, 2017; Utsch et al. 2018; Westerink 2012). Therefore, findings from investigation into the relationship between “R/S and health” are difficult to interpret. A more informative approach is taken by studies that distinguish between religiosity and spirituality and rely on the respondents’ definition of both. Here, links have been established between spirituality and a range of indicators of low mental health, such as depression, anxiety, addiction, and neuroticism (King et al. 2013; Schnell 2012; Vittengl 2018). These findings underline the necessity to distinguish between religiosity and spirituality when researching correlates of worldview positions.

With the rise of New Atheism, attention has also been drawn to the non-religious, who had been largely neglected by psychological research. Some first studies targeted and explored this group as so-called “nones”: those who, when asked to state their confession, marked none of the named religions, but ticked off “none” (Kosmin and Keysar 2009; Vernon 1968). This nomination, however, does not do justice to the variety of worldviews that may be advocated by those who are not committed to a specific religion. More recent studies suggested that different degrees of atheism and agnosticism can be held and that these are consistently linked to various beliefs and attitudes (Schnell 2015). Furthermore, individuals who share the conviction that no god or higher power exists may still hold very different worldviews, as has been shown with regard to meaning in life (Schnell and Keenan 2011), spirituality (Schnell and Keenan 2013), and several other characteristics (Keller et al. 2018).

Up to now, research on the health of atheists and other secular orientations is rare. In 2012, Weber and colleagues found 14 articles that examined levels of psychological distress among non-believers and believers. In their review, they concluded that there is “a clear correlation between strength of conviction in one’s religious (or non-religious) worldview and psychological well-being, with both the most and least religious individuals experiencing the best health” (Weber et al. 2012, p. 80). For example, Buggle and colleagues (2000) found the lowest depression rates in strong believers as well as strong atheists, compared to agnostics and less convinced believers. Galen and Kloet (2011) reported higher scores of mental well-being for individuals with higher confidence in their worldview, be it religious or atheist, relative to those who self-described as agnostics or as being unsure. This suggests that confident non-religious worldviews may have benefits similar to strong religious beliefs, by offering orientation and stability. A more recent study by Baker and colleagues (2018) also pointed in this direction. They found better mental and physical health outcomes for atheists than for other seculars and some religious traditions. Nonaffiliated theists were the least healthy.

All these findings should be interpreted with attention to the culture and context in which the studies were carried out. As demonstrated by Stavrova (2015), positive links between R/S and health are significantly stronger in countries and regions in which religiosity is more common and socially desirable. The majority of positive relationships between R/S and health have been found in the USA, where religion is much more widespread and accepted than in many European countries. Negative links between spirituality, religiosity, and health have been shown in Great Britain (King et al. 2013), Germany (Schnell 2012) and Denmark (Hvidt et al. 2017).

Cardiovascular and neuroendocrine measures reflect recent health conditions and predict future disease pathways (Chida and Steptoe 2010; Lovallo 2015). Baseline as well as reactivity and recovery measures are informative parameters. Cardiovascular reactivity is a well-established and researched concept in bio-behavioral medicine (Lovallo 2015). Elevated heart rate or blood pressure response to physical, mental, or social stress is a risk factor for future hypertension and other cardiovascular diseases (Aune et al. 2017). Exaggerated or blunted cortisol response to laboratory stress as well as a general cortisol dysregulation regarding cortisol level and variability have also been shown to predict the risk of future diseases (Dedovic and Ngiam 2015; Ennis et al. 2017; Phillips et al. 2013).

Apart from the effects of cardiovascular and neuroendocrine processes on future physical health, there is accumulating evidence for reciprocal interaction between such processes and the mind (Yan 2016). Research has established religion as a protective factor against high blood pressure (Seeman et al. 2003; Sørenson 2011). Religiosity also moderates cardiovascular reactivity (Hefti 2009; Masters et al. 2004) and recovery (Hefti 2014), which are key concepts in cardiovascular health (Lucchese et al. 2013). Spirituality, operationalized as sense of peace, on the one hand, and compassionate view of others, on the other hand, has been shown to relate to lower cortisol levels in long-term AIDS survivors (Ironson et al. 2002). Composite scores of R/S, religiosity, and frequency of prayer were found to be positively linked with lower cortisol responses to acute stressors (Tartaro et al. 2005). So far, no studies have investigated biological markers related to atheism and agnosticism.

## Material and Method

Based on the literature, we hypothesize that religiosity—but not spirituality—will be related to positive physiological health parameters in a social stress test, i.e., lower average levels of blood pressure, heart rate, and cortisol output as well as lower stress reactivity and better recovery of blood pressure, heart rate, and cortisol output. We also hypothesize that atheism will manifest in positive health parameters, in contrast to agnosticism. In line with Weber and colleagues (2012), we assume that atheists gain orientation and stability due to the decidedness of their denial of theism, while agnostics lack this decidedness. Atheism has been found to be related to a positive attitude toward science and technology, and a highly negative attitude toward religious belief and belonging, and spirituality (Schnell 2015). Agnosticism, instead, is not defined by a specific set of beliefs. A person who self-defines as agnostic expresses the assumption that nothing can be known about the existence of a god or higher power. This assumption per se is neither orienting nor stabilizing. We further assume that the strength of conviction, assessed by dimensional measures of religiosity, atheism, and existential search (representing low conviction), will influence cardiovascular and neuro-hormonal reaction to acute stress in a laboratory setting. Reactions should be better regulated when worldview conviction (religious or atheist) is high and existential search low.

### Participants

In an initial online pre-test, 205 students at an Austrian university (all of them of Caucasian background) completed a set of questionnaires. Of those who agreed to participate in a subsequent experimental study, 60 were invited. They were chosen with the aim of an as even as possible distribution of self-identification as religious, spiritual, atheist, and agnostic. All religious (*n* = 13) and atheist (*n* = 15) students from the questionnaire sample were contacted, as well as an equal number of agnostic and spiritual participants. Of those invited, 51 showed up on the allocated day of the experiment. Of these, 14% self-identified as atheist, 36% as agnostic, 20% as religious, and 30% as spiritual. The mean age was 23 years (19 to 35, SD = 4). Sixty-four percent were female.

Participants received course credits for their involvement. The study was carried out in accordance with the Declaration of Helsinki and respective ethical standards of research at the University of Innsbruck. Participation was entirely voluntary, after written informed consent. All participants were aware that they could withdraw from the study at any time. Data were anonymized from the start and handled confidentially. A debriefing took place immediately after the experiment.

### Measures

#### Questionnaires

All participants completed a set of online questionnaires, comprising the following scales: atheism (sample item: *The existence of a god/a higher power is wishful thinking.* 0–5) from the Dimensions of Secularity Inventory (DoS, Schnell 2015), centrality of religiosity (sample item: *How important is personal prayer for you?* 1–5; Huber 2008; Huber and Huber 2012), and existential search (Schnell and Geidies 2016; sample item: *As far as my worldview is concerned, I am in constant development*. 0–5). On the day of stress testing, the state version of the State-Trait-Anxiety Inventory (Laux et al. 1981) was employed to measure inter-individual differences in pre-stress anxiety. It contains 20 items (sample item: *I am tense*. 1–4). All internal consistencies are reported in Table 4.

#### Physiological Measures

**Fig. 1:**
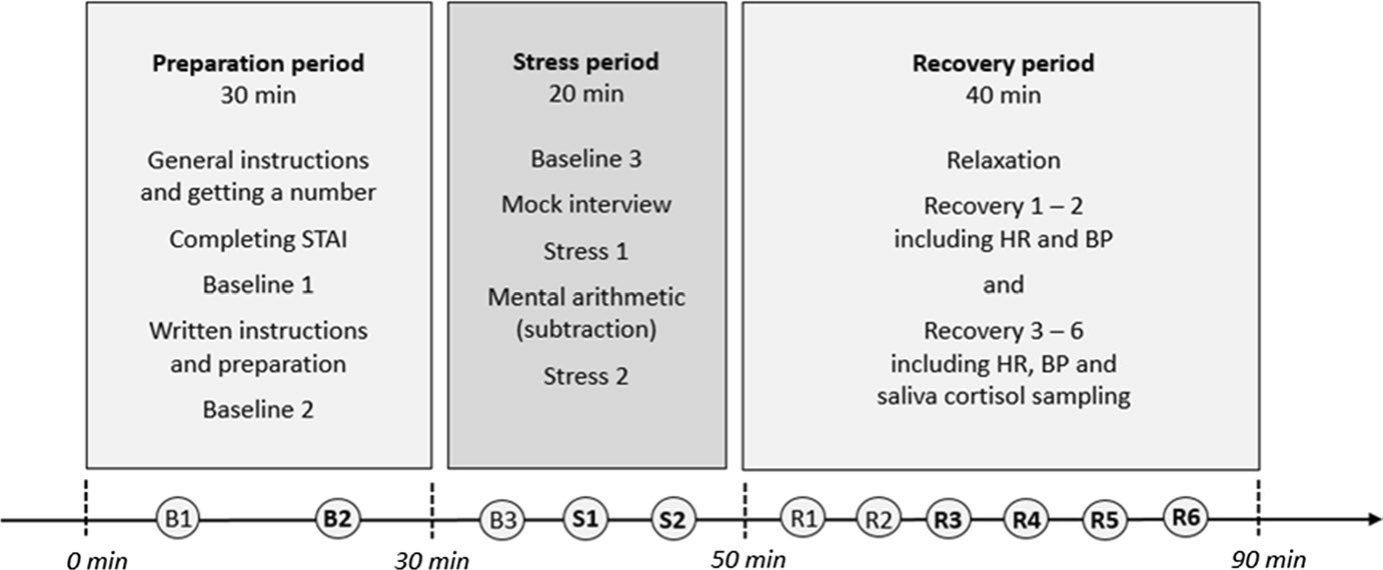
Stress protocol of the Trier Social Stress Test for Groups (TSST-G) as applied in the study

Blood pressure (BP) and heart rate (HR) were assessed with a wrist blood pressure monitor (Panasonic EW-BW10), positioned on participants’ non-dominant arm. A first baseline recording (B1, see Fig. 1) was carried out to familiarize participants with the device and to check for significantly raised blood pressure. The limit was set at 150/100 mmHg. Participants who exceeded one or both limits were excluded from further participation and advised to see their general practitioner. Due to this procedure, one person was excluded, leaving a total of 50 participants. Blood pressure was again assessed during the preparation period (B2), after transfer to the experimental setting (B3), immediately after stressors (S1, S2), and six times during the recovery period (R1–R6).

Free cortisol concentration in saliva was measured using an enzyme-linked immunosorbent assay (ELISA) kit according to the manufacturer’s instructions (Sarstedt, Nümbrecht, Germany), which required participants to saturate a cotton swab with saliva by chewing on it for a 2-minute period. Saliva samples were collected at seven points during the experiment: before the start (B2), directly after stressors (S1, S2), and four times during the recovery period (R3–R6). Samples were frozen immediately after the experiment and, after a storage period of up to one week, biochemically analyzed at the Division of Biological Chemistry, Medical University of Innsbruck).

### Procedure

Participants underwent the Trier Social Stress Test for Groups (TSST-G; von Dawans et al. 2011). The TSST-G combines high levels of socio-evaluative threat and uncontrollability to induce psychological stress in a group format. Participants had been told that they would be taking part in an “experiment on worldview and stress management.” They had also been instructed to abstain from caffeine, alcohol, exercise, and smoking three hours prior to the testing. Groups consisted of up to five participants. To guarantee equal conditions, testing had been planned to take place during lunch time (between 11 am and 2 pm). Due to time constraints of the participants, however, seven people took the test before 11 am and eight after 2 pm. (Time of testing was statistically controlled in the analyses.) As shown in Fig. 1, the procedure included a preparation period (30 min), the stress task itself (20 min), and a recovery period (40 min).

*Preparation period (room A):* After receiving detailed instructions, participants signed informed consent. Each of them was then assigned a number from 1 to 5 (or lower, if less than five participants were present). They then completed the State-Trait-Anxiety Inventory (STAI), and two baseline measures of heart rate (HR) and blood pressure (BP; systolic and diastolic, SBP and DBP) were taken (B1, B2). Along with B2, the first saliva collection was acquired.

In between B1 and B2, participants received written instructions about the first task. They were asked to prepare a presentation of themselves for a mock job interview, which they would have to give in two minutes of free speech, in front of a jury. They had ten minutes for preparation; they were allowed to use paper and pencil but could not take their notes to the presentation.

*Stress period (room B):* After the preparation phase of 10 min, all participants were guided to room B and had to sit in a row in front of a committee consisting of a man and a woman, dressed in white lab coats, who sat behind a table that was framed by two conspicuous video-cameras (these were powered, but—unknown to the participants—paused). The two members of the evaluation committee were trained to withhold any verbal and non-verbal feedback. Participants were separated by mobile walls that restricted any eye contact and social interaction with the other participants. Another baseline measure of HR and BP was taken (B3). The committee then called on each of the numbered participants in random order to come to the fore and start their speech. Whenever a participant finished their presentation in less than two minutes, the committee responded in a standardized way: “You still have some time left. Please continue!” After all participants had given their two-minute speech, they were called upon again, one by one, and asked to serially subtract the number 16 from a given number > 4800 for a duration of one minute, as quickly as possible. Each participant received an individual starting number to avoid learning effects. If they made a mistake, they had to restart at their personal number. After the mock interview and the arithmetic task, HR, BP, and saliva cortisol were measured again (S1, S2).

*Recovery period (room B/C):* Participants were instructed to actively try to relax. They should do this in the way they were used to in their private lives, but without using any devices. Within this time period, six recovery measures were taken for HR and BP (R1–R6) and four recovery measure for saliva cortisol (R3–R6).

After the recovery period, participants were asked to comment in writing on their experience of the experiment and their chosen method of relaxation in a short questionnaire. Finally, they were offered some sweets and debriefed about the test, with particular emphasis on the fact that their performance had not been documented, nor recorded by the camera.

### Data Analysis

Data analyses were carried out with IBM SPSS Statistics 22. Several variables were skewed, but none beyond ± 2.00. In line with George and Mallery (2010), we therefore used parametric methods. Levene’s test was used to test for homogeneity of variance in all dependent variables. Reported results were corrected by the Greenhouse–Geisser procedure when the assumption of sphericity was violated.

Preliminary analyses tested for potential covariates, including time of day of participation, exercising before participation, hours of sleep in previous night, being postmenopausal (women), suffering from an acute or chronic illness, and hormone treatments (estrogen, testosterone, DHEA, and hormonal contraception). Because their correlations with cortisol measures exceeded *r* > .20, the following variables were included as covariates in cortisol analyses: use of hormonal contraceptives and hours of sleep in the previous night. In blood pressure analyses, covariates were sex, use of hormonal contraceptives, and suffering from an acute or chronic disease (*r* > .20). Due to the small sample size and ensuing low statistical power, findings are reported both with and without covariates. Bootstrapping (1000 samples) was used for all analyses, and 95%/90% BCa confidence intervals are reported. To prevent further substantial reduction in statistical power of rejecting an incorrect H_0_, no Bonferroni corrections were employed and readers are asked to evaluate the findings’ relevance by focusing on effect sizes and confidence intervals (cf., Nakagawa 2004).

The following parameters were used as indicators of cardiovascular stress regulation: baseline (mean B1 to B3), stress reactivity (mean S1, S2 minus mean B1 to B3), stress recovery (mean R1 to R6 minus mean B1 to B3, with high scores indicating insufficient recovery), and average scores across all measures. Cortisol stress regulation was assessed by baseline, AUC_g_ (area under the curve with respect to ground), stress recovery (personal minimal concentration of cortisol; cf. Miller et al. 2018), and average values (cf. Khoury et al. 2015) across all measures.

## Results

### Pre-stress State Anxiety

Pre-stress anxiety was *M* = 37.24 (SD = 7.60). This is comparable to the original German norm sample scores (*M*_male_ = 36.83 to *M*_female_ = 38.08, SD = 9.82 to 10.29; Laux et al. 1981), indicating regular state anxiety levels. No differences between the four self-identified groups (agnostic, atheist, religious, and spiritual) were established (*F*(3, 46) = 0.18, *p* = .91, *η*^2^ = .01).

### Physiological Effects of the TSST-G

Three one-way repeated measures ANOVAs across all participants demonstrated significant time effects for SBP, *F*(6.85, 336) = 56.49, *p* < .001, *η*^2^ = .54), DBP, *F*(4.99, 244) = 13.97, *p* = < .001, *η*^2^ = .22), and heart rate, *F*(5.58, 274) = 26.23, *p* < .001, *η*^2^ = .35). This suggests that for cardiovascular measures, the exposure to socio-evaluative stress did succeed in manipulating relevant scores (see Table 1).

**Table 1:** Means and standard deviations of cardiovascular measures and salivary cortisol at all measurement points, total sample (*N* = 50)

	SBP	DBP	HR	SC
Baseline 1	119.10 (11.42)	81.80 (9.18)	79.40 (10.86)	
Baseline 2	123.92 (11.53)	86.38 (8.67)	86.80 (12.83)	16.46 (23.51)
Baseline 3	138.16 (12.67)	95.36 (7.74)	81.24 (14.41)	
Stress 1	140.62 (11.70)	96.54 (8.57)	95.00 (17.91)	13.27 (17.56)
Stress 2	138.76 (14.19)	95.02 (15.68)	92.28 (17.79)	10.32 (12.20)
Recovery 1	127.82 (11.98)	91.84 (7.14)	81.38 (12.59)	8.91 (7.79)
Recovery 2	125.84 (10.12)	89.88 (7.71)	79.08 (12.03)	
Recovery 3	126.96 (11.14)	88.02 (11.73)	82.50 (14.33)	8.35 (9.14)
Recovery 4	127.32 (11.33)	86.44 (16.31)	81.56 (11.08)	7.79 (7.05)
Recovery 5	125.84 (10.35)	89.80 (10.87)	79.96 (10.58)	
Recovery 6	124.30 (10.75)	89.20 (8.30)	79.06 (9.88)	7.67 (5.56)

SBP = systolic blood pressure; DBP = diastolic blood pressure; HR = heart rate per minute. SC = salivary cortisol

There was also a repeated measures effect of the TSST on salivary cortisol, *F*(2.66, 130) = 3.46, *p* = .02, *η*^2^ = .07). However, cortisol output did not rise with the stressors but was highest at baseline, suggesting a high degree of anticipatory activation (see Table 1).

### Worldview and Cardiovascular Stress Response: Group Differences

We first tested the assumption that self-identified religious and atheist participants would show lower baseline and average blood pressure and heart rate scores, lower cardiovascular reactivity to the stress tasks and better stress recovery than spiritual and agnostic participants. Three multivariate analyses of (co)variance were carried out for baseline, average scores, and stress recovery. Due to the necessity of additionally controlling for specific baseline scores, three univariate analyses of covariance were carried out for stress reactivity. Table 2 displays descriptive statistics for cardiovascular parameters, for the total sample as well as for the four subgroups of self-identified atheist, agnostic, religious, and spiritual participants.

**Table 2:** Means and standard deviations of cardiovascular measures for total sample and four subgroups, and estimated means and standard errors for four subgroups controlled for covariates

	Total sample	Atheist	Agnostic	Religious	Spiritual
SBP baseline^a^	121.51 (11.03)	**127.79** **(16.91)**^**x**^	120.61 (10.24)	**115.75** **(7.80)**^**xy**^	**123.50** **(9.38)**^**y**^
With covariates^e^		125.72 (3.66)	118.94 (2.27)	118.55 (3.17)	124.61 (2.45)
SBP stress reactivity^b^	20.59 (10.34)	**27.57** **(3.78)**^**x**^	19.77 (2.30)	**15.64** **(3.18)**^**x**^	21.61 (2.53)
With covariates^e^		**27.76** **(3.97)**^**x**^	19.87 (2.47)	**15.42** **(3.43)**^**x**^	21.55 (2.67)
SBP stress recovery^c^	4.84 (7.42)	5.19 (8.71)	6.96 (8.06)	3.98 (5.58)	2.69 (7.09)
With covariates^e^		5.36 (2.94)	**7.23** **(1.83)**^**x**^	3.65 (2.55)	**2.51** **(1.97)**^**x**^
SBP average^d^	128.97 (9.77)	**136.31** **(12.31)**^**xy**^	**129.27** **(10.29)**^**yz**^	**122.05** **(7.75)**^**xzw**^	**129.79** **(6.33)**^**w**^
With covariates^e^		**134.55** **(3.16)**^**xy**^	**127.90** **(1.97)**^**y**^	**124.39** **(2.74)**^**xz**^	**130.70** **(2.12)**^**z**^
DBP baseline^a^	87.85 (7.28)	91.00 (6.09)	87.91 (8.49)	87.00 (6.12)	86.87 (7.17)
With covariates^e^		91.39 (2.84)	87.85 (1.76)	86.67 (2.46)	86.98 (1.90)
DBP stress reactivity^b^	13.98 (9.73)	10.71 (8.86)	14.53 (11.05)	11.80 (6.88)	16.30 (10.20)
With covariates^e^		11.74 (3.98)	14.70 (2.43)	11.33 (3.40)	15.93 (2.62)
DBP stress recovery^c^	1.35 (6.36)	2.79 (7.06)	1.92 (6.07)	0.18 (5.47)	0.78 (7.33)
With covariates^e^		2.52 (2.17)	2.25 (1.35)	0.23 (1.88)	0.47 (1.45)
DBP average^d^	90.03 (6.95)	93.74 (8.41)	90.35 (8.41)	88.34 (5.02)	89.03 (6.79)
With covariates^e^		94.12 (2.78)	90.56 (1.73)	87.85 (2.42)	88.92 (1.86)
HR baseline^a^	82.48 (11.45)	78.19 (11.93)	82.83 (15.08)	81.63 (7.31)	84.62 (8.53)
With covariates^e^		80.98 (4.15)	84.06 (2.58)	78.02 (3.60)	84.25 (2.78)
HR stress reactivity^b^	14.24 (12.86)	**23.63** **(4.62)**^**x**^	11.41 (2.85)	**8.51** **(3.82)**^**xy**^	**17.07** **(3.14)**^**y**^
With covariates^e^		**24.14** **(4.80)**^**x**^	11.68 (2.99)	**7.99** **(4.23)**^**xy**^	**16.86** **(3.22)**^**y**^
HR stress recovery^c^	− 1.89 (5.70)	− 1.17 (4.59)	− 2.19 (6.08)	− 2.85 (5.22)	− 1.23 (6.39)
With covariates^e^		− 2.21 (2.21)	− 2.79 (1.37)	− 1.60 (1.92)	− 0.86 (1.48)
HR average^d^	83.48 (11.24)	80.79 (12.39)	83.01 (14.22)	81.49 (8.28)	86.62 (8.35)
With covariates^e^		83.20 (4.08)	84.00 (2.53)	**78.33** **(3.54)**^**x**^	**86.42** **(2.73)**^**x**^

For sign. ps and BCa CI, see supplementary material, Table A

SBP = systolic blood pressure; DBP = diastolic blood pressure; HR = heart rate per minute

^a^Baseline (*M*_B1 to B3_)

^b^Stress reactivity (*M*_S1 to S2_) minus baseline; controlled for baseline

^c^Stress recovery (*M*_R1 to R6_) minus baseline

^d^Mean of all measures

^e^Estimated means and standard errors evaluated at covariates sex = 1.36 (*f* = 1, *m* = 2), use of hormonal contraceptives = 0.36 (0/1), suffering from an acute or chronic disease = 0.12 (0/1). Stress reactivity data additionally evaluated at baseline SBP = 121.51, baseline DBP = 87.85, baseline heart rate = 82.48

^xx, yy, zz, ww^Significant bootstrapped pairwise comparisons are given in bold

When covariates were included, hypothesized differences were established between religious and spiritual participants, with the former showing better stress responses in SBP and HR average scores and HR stress reactivity. Contrary to our expectation, atheists’ stress was higher than religious participants’ in SBP and HR stress reactivity and average SBP, and higher than agnostics’ in average SBP. In the one case of SBP stress recovery, spiritual participants showed better scores than the other groups, significantly so in comparison with agnostics. When multiple comparisons were carried out without controlling for covariates, the data indicated lower stress among religious than spiritual participants with regard to baseline and average SBP, and HR stress reactivity. Again, atheists showed higher stress than religious participants in baseline and average SBP, as well as in SBP and HR stress reactivity, and also higher average SBP than agnostics. In sum, findings indicate especially low stress among religious participants, higher stress among spiritual participants, and especially high parameters among atheists.

### Worldview and Cortisol Output: Group Differences

**Fig. 2:**
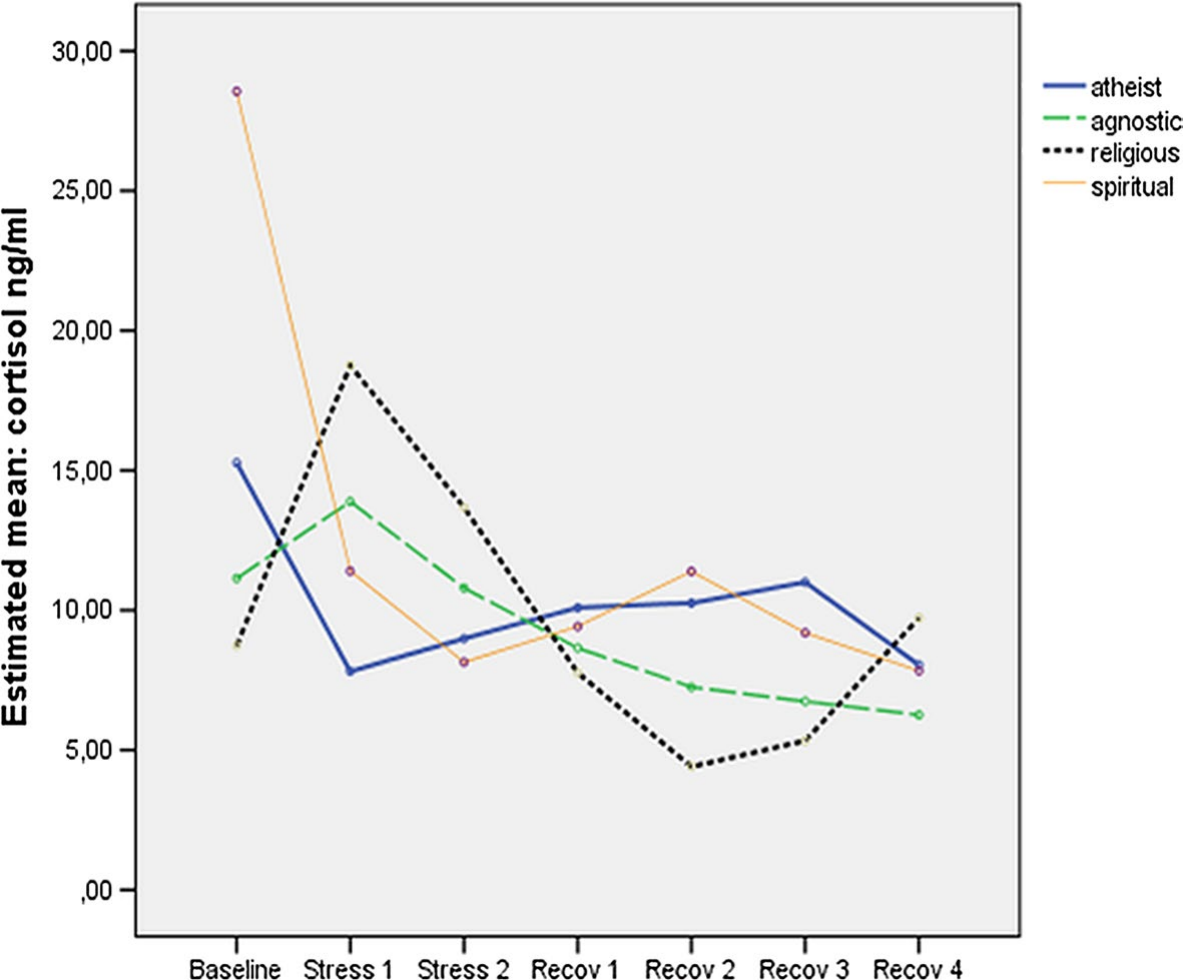
Cortisol output (estimated means, controlled for covariates) at seven measurement points for four worldview groups

Descriptive statistics of cortisol measures suggested differential progression of cortisol output over the seven measurement points for the four worldview groups. As illustrated in Fig. 2, only religious and agnostic participants showed the expected increase of cortisol output after stressors 1 and 2. In contrast, the highest mean cortisol scores among both spiritual and atheist participants were recorded at baseline, followed by a sharp drop of cortisol output. Miller et al. (2013) recommend classifying those participants as non-responders who show a less than 1.5 nmol/l (equivalent to .05 ng/mL) baseline-to-peak increase. When applying this criterion to the present sample, as many as 70% would have to be identified as non-responders. Calculation of stress reactivity is thus not feasible. Some indices had large standard errors, which is a recognized phenomenon in the HPA literature (Atkinson et al. 2013), suggesting intra- and inter-individual variability.

Group differences—both with and without covariates—were significant for baseline cortisol scores, only (see Table 3). Spiritual participants’ baseline cortisol was higher than that measured in religious and agnostic participants.

**Table 3:** Means and standard deviations of cortisol output for total sample and four subgroups, estimated means and standard errors for four subgroups controlled for covariates

	Total sample	Atheist	Agnostic	Religious	Spiritual
SC baseline	16.46 (23.51)	17.46 (33.65)	**11.66**^**x**^ **(19.92)**	**7.24**^**y**^ **(6.30)**	**27.91**^**xy**^ **(26.64)**
With covariates^a^		14.66 (8.90)	**11.22**^**x**^ **(5.42)**	**9.33**^**y**^ **(7.42)**	**28.35**^**xy**^ **(5.91)**
Average	10.40 (6.40)	10.59 (10.33)	9.28 (4.11)	9.77 (6.10)	10.06 (6.94)
With covariates^a^		9.85 (2.54)	9.29 (1.55)	10.12 (2.12)	12.16 (1.68)
AUC_g_^b^	891.84 (592.68)	853.03 (877.58)	804.43 (420.37)	854.98 (646.57)	1039.42 (617.41)
With covariates^a^		794.69 (237.06)	805.53 (144.40)	882.10 (197.75)	1047.24 (157.31)
Recovery^c^	30.06 (24.24)	27.44 (30.51)	26.89 (22.12)	31.72 (25.63)	33.97 (24.64)
With covariates^a^		25.78 (9.79)	27.16 (5.96)	32.12 (8.16)	34.16 (6.49)

For sign. ps and BCa CI, see supplementary material, Table A

^a^Estimated means and standard errors evaluated at covariates hours of sleep in previous night = 7.63 and use of hormonal contraceptives = 0.36

^b^Area under the curve with respect to the ground

^c^Personal minimal concentration of cortisol

^xx, yy^Significant bootstrapped pairwise comparisons are given in bold

### Dimensional Worldview Predictors of Cardiovascular and Cortisol Response

The following analyses examined effects of the intensity of commitment to atheism and religiosity, and the degree of existential search. They thus tested the hypothesis that the strength of specific worldview convictions should have an impact on health parameters, such as cardiovascular and cortisol levels in a context of acute social stress. Table 4 reports internal consistencies and bivariate and partial correlations, controlling for the relevant covariates as described above.

**Table 4:** Internal consistencies (Cronbach alpha) of worldview scales; bivariate and partial correlations with cardiovascular and cortisol scores

	Atheism	Religiosity	Existential search
Cronbach alpha	.94	.76	.68
DBP baseline	.11	− .22	.00
Partial^a^	.07	− .19	.06
DBP stress reactivity^b^	− .06	.06	.17
Partial^a,b^	− .07	.06	.22
DBP stress recovery	− .06	.07	.27*
Partial^a^	− .01	− .02	.17
DBP average	.06	− .18	.16
Partial^a^	.06	− .19	.17
SBP baseline	.17	− .19	.13
Partial^a^	− .04	.07	.13
SBP stress reactivity^b^	.20	− .29*	.28*
Partial^a,b^	.20	− .31*	.30*
SBP stress recovery	.14	− .18	.17
Partial^a^	.17	− .23	.14
SBP average	.28*	− 34**	.27*
Partial^a^	.10	− .12	.28*
HR baseline	− .14	.07	.17
Partial^a^	.00	− .05	.28*
HR stress reactivity^b^	.11	− .15	.24*
Partial^a,b^	.09	− .14	.29*
HR stress recovery	.08	− .09	.26*
Partial^a^	− .02	.04	.20
HR average	− .12	.05	.30*
Partial^a^	.00	− .05	.40*
SC baseline	− .01	− .02	.37**
Partial^c^	− .03	− .02	.34*
SC AUC_g_	− .03	− .02	.30*
Partial^c^	− .05	.00	.30*
SC stress recovery	− .07	.06	.22
Partial^c^	− .07	.08	.23
SC average	− .02	− .06	.33*
Partial^c^	− .03	− .02	.33*

Significant values are given in bold

Sign. BCa 90% CIs in Supplementary Material, Table B

*p < .05; **p < .01, one-sided

^a^Partial correlations, controlling for sex, use of hormonal contraceptives, and suffering from an acute or chronic disease

^b^Stress reactivity data additionally controlling for baseline DBP, baseline SBP, and baseline heart rate, resp

^c^Partial correlations, controlling for hours of sleep in previous night and use of hormonal contraceptives

The results lend little support to our hypothesis that reported degrees of atheism and religiosity would predict biological markers. Expected significant findings only showed for SBP, in that religiosity predicted lower reactivity scores (with and without controlling for covariates). Without covariates, average SBP decreased with religiosity but—contrary to our hypothesis—increased with atheism. The effects disappeared when covariates were included.

In line with our hypothesis, associations between existential search and stress parameters were positive and marked. In uncorrected as well as partial correlations, existential search predicted higher SPB average scores and higher SBP stress reactivity, higher HR average and reactivity scores, as well as higher cortisol baseline, average, and AUC_g_ scores.

To explore this prominent role of existential search further, a univariate analysis of covariance was conducted to test for differences in existential search between the four self-identified worldview groups, controlling for sex. A general group effect was established (*F*(3, 45) = 2.40, *p* = .04, *η*^2^ = .14). Bootstrapped pairwise comparisons indicated that spiritual participants had higher scores in existential search than atheist (BCa 95% CI [.002, 1.52]), religious (BCa 95% CI [.001, 1.33]), and agnostic (BCa 95% CI [.012, 1.16]) participants.

## Discussion

The aim of the present study was to test the effects of specific worldviews on dealing with social stress, measured by cardiovascular parameters and cortisol output in a sample of 50 healthy students undergoing standardized social stress testing. Based on the literature (e.g., Hill and Pargament 2008; Johnstone et al. 2008; Koenig 2012; Koenig et al. 2012; Vance et al. 2008; Weber et al. 2012), we expected a positive impact of religiosity and convinced atheism on stress regulation, while this was not expected for agnosticism, and assumed to be the contrary for spirituality (King et al. 2013; Schnell 2012; Vittengl 2018).

### Main Findings

The analyses showed group differences between religious, atheist, and spiritual participants. These were mostly visible in systolic blood pressure and heart rate parameters, and in baseline cortisol measures. As expected, religious participants showed low cardiovascular stress responses. Spiritual participants had higher average SBP and heart rates than religious participants. Their baseline cortisol scores were higher than those of religious, but also agnostic participants, lending support to our assumption that spirituality should be distinguished from religiosity.

Contrary to our expectation, self-identification as atheist was not associated with an advantage in dealing with social stress. Atheists’ stress responses were substantially higher than those shown by self-identified religious participants. Self-ascribed atheism may (theoretically) suggest an orientating worldview, but it does not appear to be a predictor of healthy stress regulation.

While the Trier Social Stress Test raised cardiovascular stress levels, this was not the case for cortisol output. A large number of participants showed an atypical cortisol profile, with high cortisol output at baseline, followed by a decrease during stress tasks. Descriptive analyses of the four worldview groups revealed that two of them, the religious and agnostic groups, showed the expected profile, with an increase of cortisol output after the stress tasks. Spiritual and atheist participants, on the other hand, had the highest cortisol output at baseline. This suggests an anticipatory stress response, awaiting the challenges of an “experiment on worldview and stress management.” This finding might be another indicator of particularly high stress reactivity, as had been hypothesized for the spiritual, and was empirically demonstrated (see above) for atheists.

Results derived from dimensional worldview analyses contributed further to the assumption that worldview stability might serve as a foundation for healthy stress regulation. Existential search in particular showed strong associations with physiological measures, both cardiovascular (SBP and HR) and endocrine (SC). Operationalizing an open, uncertain worldview, existential search was related to higher average and baseline stress parameters as well as to higher stress reactivity. With respect to self-identification, existential search was highest in the spiritual group. This gives further credence to the hypothesis that spirituality may not be linked to the same health benefits as has been shown for religiosity (King et al. 2013; Schnell 2012). It also highlights the necessity to differentiate between spirituality and religiosity.

Measures of centrality, or degree, of atheism and religion were of less informative value. With regard to atheism, correlational analyses did not support the assumption that (a high commitment to) atheism might be beneficial for health in a social stress context. Results for committed religiosity were slightly more supportive, since significant associations with systolic blood pressure were found. They pointed in the expected direction, suggesting an assumed stress buffering capacity of religiosity.

### Limitations

The main limitation of this study is its sample size. Although an average size for Trier Social Stress Test Studies, study group numbers were especially critical in the first part of the analyses, with low statistical power to detect group differences between self-identified atheist, agnostic, religious, and spiritual participants. The results should thus be viewed as preliminary, and future studies with a focus on secular worldviews are required to replicate or correct our findings, and expand our knowledge on worldviews beyond religion.

### Conclusion

The present study contributes to the literature on the mind-body connection by lending support to the assumption that facets of worldview may predict ways of handling acute social stress, as measured by physiological markers. In particular, existential search—a concept of (negative) worldview security—showed a number of robust associations with physiological stress parameters, indicating an elevated stress response. It might be a crucial element to understand why some worldviews—as, e.g., religiosity—seem to be beneficial with regard to mental and physical health, while this does not hold for self-ascribed spirituality. Spirituality, in the current study, was characterized by the highest degree of existential search. Following this, the association between religiosity and positive health could be due to the relative clarity and stability of religious worldviews, in contrast to spiritual, agnostic, and—probably—also atheist outlooks. Contrary to our expectation, neither a self-identification as atheist nor a strong commitment to atheism was related to better health outcomes; rather, there was some evidence for the opposite.

## Electronic supplementary material

Below is the link to the electronic supplementary material.

Supplementary material 1 (DOCX 14 kb)
