# Supplementary material for: Worldview Under Stress: Preliminary Findings on Cardiovascular and Cortisol Stress Responses Predicted by Secularity, Religiosity, Spirituality, and Existential Search
Source: J Relig Health. 2020 Mar 27;59(6):2969–89. doi: 10.1007/s10943-020-01008-5 (PMC7677289; doi:10.1007/s10943-020-01008-5)
Supplement: Supplementary file 1 — Supplementary material 1 (DOCX 14 kb) [file 10943_2020_1008_MOESM1_ESM.docx]

Supplementary Material

Table A. Significance Levels, Mean Differences and BCa Confidence Intervals for Significant Multiple Comparisons

|  | Covariates yes/no | Groups | p(one-sided) | Mean difference | BCa 90% CI |
| --- | --- | --- | --- | --- | --- |
| SBP baseline | no | Atheist – Religious  Spiritual – Religious | .014  .042 | 12.04  7.75 | 3.17/20.90  15.09/0.41 |
| SBP stress reactivity | no | Atheist – Religious | .018 | 11.93 | 2.06/20.45 |
| SBP stress recovery ^c)^ | no | Agnostic – Spiritual | .045 | 4.72 | .015/9.28 |
| SBP average ^d)^ | no | Atheist – Agnostic  Atheist – Religious  Spiritual – Religious  Agnostic – Religious | .045  .002  .022  .025 | 7.04  14.27  7.74  7.23 | 0.23/13.85  6.73/21.80  1.50/13.98  1.20/13.25 |
| HR stress reactivity^b)^ | no | Atheist – Religious  Spiritual – Religious | .03  .03 | 15.13  8.56 | 2.93/26.37  2.23/15.21 |
| SC Baseline | no | Spiritual – Agnostic  Spiritual – Religious | .024  .015 | 16.26  20.67 | 2.90/29.62  5.07/36.27 |
| SBP stress reactivity |  | Atheist – Religious | .023 | 12.34 | 0.92/22.83 |
| SBP average |  | Atheist – Agnostic  Atheist – Religious  Spiritual – Religious | .038  .012  .035 | 6.63  10.14  6.31 | 0.50/12.76  2.83/17.45  0.57/12.05 |
| HR stress reactivity |  | Atheist – Religious  Spiritual – Religious | .030  .049 | 16.15  8.87 | 1.07/29.65  0.24/17.81 |
| HR average |  | Spiritual – Religious | .037 | 8.09 | 0.70/15.48 |
| SC Baseline |  | Spiritual – Agnostic  Spiritual – Religious | .018  .025 | 17.13  19.02 | 3.64/30.62  3.18/34.86 |

Table B. Bivariate/Partial Correlation and BCa 90% CI for Significant Correlations

| Worldview Scale | Biological Marker | Partial yes/no | Correlation Coefficient | BCa 90% CI |
| --- | --- | --- | --- | --- |
| Existential Search | DBP stress recovery | no | .27 | .024/.484 |
| Existential Search | HR stress recovery | no | .26 | .052/.454 |
| Existential Search | SBP average | no | .27 | .062/.464 |
| Existential Search | HR average | no | .30 | .114/.508 |
| Existential Search | SBP stress reactivity | no | .28 | .036/.504 |
| Existential Search | HR reactivity | no | .24 | .003/.476 |
| Existential Search | SC baseline | no | .37 | .219/.515 |
| Existential Search | SC AUC _g_ | no | .30 | .074/.501 |
| Existential Search | SC average | no | .33 | .121/.504 |
| Religiosity | SBP average | no | -.34 | -.531/-.118 |
| Religiosity | SBP stress reactivity | no | -.29 | -.526/-.023 |
| Atheism | SBP average | no | .28 | .044/.491 |
| Existential Search | SBP stress reactivity | yes | .30 | .027/.512 |
| Existential Search | HR stress reactivity | yes | .29 | .005/.553 |
| Existential Search | HR baseline | yes | .28 | .080/.460 |
| Existential Search | SBP average | yes | .28 | .029/.508 |
| Existential Search | HR average | yes | .40 | .211/.575 |
| Existential Search | SC baseline | yes | .34 | .167/.503 |
| Existential Search | SC average | yes | .33 | .120/.505 |
| Existential Search | SC AUC _g_ | yes | .30 | .102/.519 |
| Religiosity | SBP stress reactivity | yes | -.31 | -.531/-.070 |
